# Molecular Engineering of Binder for Improving the Mechanical Properties and Recyclability of Energetic Composites

**DOI:** 10.3390/nano13061087

**Published:** 2023-03-17

**Authors:** Jing Yang, Xin Zhou, Xiaomu Wen, Gazi Hao, Lei Xiao, Guangpu Zhang, Wei Jiang

**Affiliations:** 1 National Special Superfine Powder Engineering Technology Research Center, Nanjing University of Science and Technology, Nanjing 210094, China; 2 Science and Technology on Transient Impact Laboratory, Research Institute of China Ordnance Industries, Beijing 102202, China

**Keywords:** zigzag structure, disulfide exchange, polymer binder, nanocomposites, recyclability

## Abstract

Mechanical properties and reprocessing properties are of great significance to the serviceability and recyclability of energetic composites. However, the mechanical robustness of mechanical properties and dynamic adaptability related to reprocessing properties are inherent contradictions, which are difficult to optimize at the same time. This paper proposed a novel molecular strategy. Multiple hydrogen bonds derived from acyl semicarbazides could construct dense hydrogen bonding arrays, strengthening physical cross-linking networks. The zigzag structure was used to break the regular arrangement formed by the tight hydrogen bonding arrays, so as to improve the dynamic adaptability of the polymer networks. The disulfide exchange reaction further excited the polymer chains to form a new “topological entanglement”, thus improving the reprocessing performance. The designed binder (D2000-ADH-SS) and nano-Al were prepared as energetic composites. Compared with the commercial binder, D2000-ADH-SS simultaneously optimized the strength and toughness of energetic composites. Due to the excellent dynamic adaptability of the binder, the tensile strength and toughness of the energetic composites still maintained the initial values, 96.69% and 92.89%, respectively, even after three hot-pressing cycles. The proposed design strategy provides ideas for the design and preparation of recyclable composites and is expected to promote the future application in energetic composites.

## 1. Introduction

Energetic composites such as solid rocket propellants and polymer binder explosives are usually composed of ultra-high content nano explosive crystals (more than 80 wt%) and small amount of polymer binders, which are widely used in military and civil environments [1,2]. In the processes of storage, transportation and usage, energetic composites with less strength and toughness will inevitably crack or be damaged due to external shock, temperature, humidity or other stimuli, further damaging the stability and security of the weapon systems [3]. In addition, the scrapping of energetic composites with cracks undoubtedly increases environmental consumption and economic losses. Strong and tough mechanical properties could help energetic composites resist external impacts or shocks. For damaged energetic composite materials, reprocessing may be an economic and environmental treatment method. Therefore, improving the mechanical properties of energetic composites and endowing them with convenient reprocessing properties are the key methods of solving the above problems. Among the reinforced nanocomposites, graphene nanoplatelet, carbon nanotubes and carbon fibers are usually responsible for reinforcing the matrix and have been widely used in engineering applications [4,5,6]. However, in the field of nano-energic composites, in order to ensure the energy level, energetic crystals account for more than 80% of it, leaving little room for the addition of reinforcing fillers.

Polymer binder is one of the materials used for energetic composites, mainly for bonding and endowing materials with mechanical properties [7]. For balancing the mechanical properties and improving the reprocessing performance of energetic composites, substantial efforts have focused on the molecular engineering of polymers [8,9,10]. However, the ultra-high filler will inevitably reduce the interface contact effect, thus intensifying the contradiction between tensile strength, toughness and reprocessing. The mechanical properties after this process compromise only 1.20~1.36 MPa [11,12]. The introduction of multiple hydrogen bonds into polymers is a common approach to enhance the mechanical properties [13,14,15]. Typically, acyl semicarbazides (ASCZ) could be used in trimers, tetramers or even larger hydrogen-bonded aggregates [16], thus forming robust supramolecular networks to improve the strength and toughness of materials [17,18]. However, it is still a challenge to design binders that could endow energetic composites with strong and tough mechanical properties and an excellent reprocessing performance. This is mainly due to the tightly packed hydrogen bonding arrays increasing the energy barrier of aggregate dissociation, which restricts the dynamic energy required for the reprocessing of materials. Reducing the hydrogen bond density is a common means to balance the mechanical properties and dynamic adaptability, but at the expense of the strength and toughness of materials [19]. Therefore, more molecular engineering strategies should focus on how to improve dynamic adaptability, while constructing robust supramolecular networks.

For the linear symmetric arrangement of urea-based structures, there is a partial crystallization behavior, which improves the mechanical properties, but leads to poor dynamic adaptability [20]. The zigzag arrangement of hydrogen bonds can destroy the regularity of polymer chains and promote the exchange of hydrogen bonds [21]. On the other hand, the disulfide bond as a weak dynamic covalent bond could be reformed and exchanged at a suitable temperature [22,23], thus endowing materials with self-healing [24,25] and reprocessing properties [26]. In addition, the dynamic exchange of disulfide bonds is conducive to the reorganization of entangled polymer chains [27], further improving the toughness of materials [28]. Thus, reasonably designed disulfide-based supramolecular networks with this unique structure could improve dynamic adaptability and mechanical robustness simultaneously.

**Figure 1:**
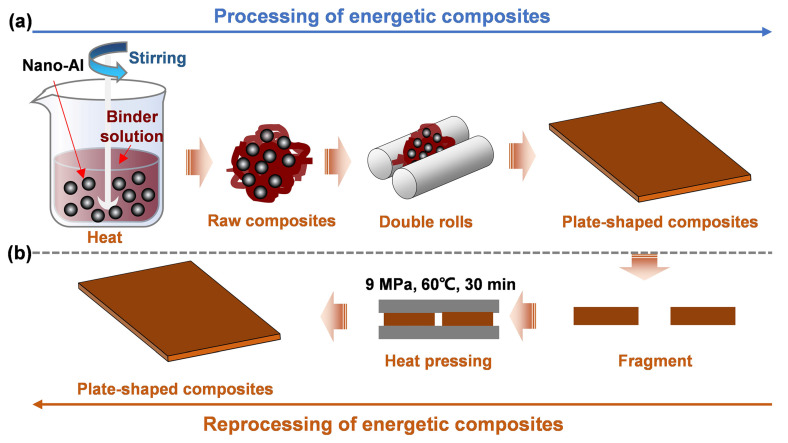
(**a**) Preparation process and (**b**) reprocessing process of D2000-ADH-SS/Al.

In this paper, we proposed a new molecular engineering strategy to prepare a polymer binder, polyetheramines-adipohydrazide-2,2′-diaminodiphenyl disulfide (D2000-ADH-SS), for further processing with nano-aluminum powder into energetic composites (D2000-ADH-SS/Al) (Figure S1 and Figure 1a). The multiple hydrogen bonds derived from ASCZ motifs (Figure S2) improved the robustness of the polymer networks. The zigzag structures broke the regular arrangement of polymers, promoting the dynamic exchange of hydrogen bonds. The introduction of aromatic disulfides further improved the toughness and recyclability of materials. Nano-aluminum powder was then embedded into the polymer networks of D2000-ADH-SS to prepare the energetic composites. D2000-ADH-SS endowed the composites with good mechanical properties. In addition, for the shredded D2000-ADH-SS/Al fragments, the dynamic exchange of disulfide bonds promoted the reconstruction of topological entanglement at the notch, thus repairing the damaged network, and finally producing D2000-ADH-SS/Al with an excellent reprocessing performance (Figure 1b).

## 2. Materials and Methods

### 2.1. Materials

Polyetheramines (D2000, Mn: ~2000), isophorone diisocyanate (IPDI, 99%), 2,2′-diaminodiphenyl disulfide (SS, 98%) and *N*,*N*-dimethylformamide (99.8%) were purchased from Macklin Reagent (Shanghai Chemical Industry Park, Shanghai, China). Methanol (99.5%) was obtained from Aladdin (No. 196 Xinjingqiao Road, Pudong, Shanghai, China). Adipohydrazide (ADH) was obtained from Bidepharm (the National University Science Park of University of Shanghai for Science and Technology, Shanghai, China). Viton (F2603) was purchased from Guangzhou Endi Plastic Products Co., Ltd. (Dongguan Changping Dajingjiu Plastic Industrial Park, Dongguan, China), which was prepared by copolymerization of 80 mol% vinylidene fluoride and 20 mol% hexafluoropropylene. Nano-Al (APS: 50 nm) was obtained from Shanghai ST-nano Science & Technology Co., Ltd. (Shanghai Jinshan Fengjing Industrial Park, Shanghai, China). All reagents were of analytical grade and were not further purified during use.

### 2.2. Synthesis of D2000-ADH-SS Binder

First, D2000 (2.50 g) and IPDI (1.11 g) were put into a round bottom flask, subjected to with magnetic stirring and left to react for 3 h in a nitrogen atmosphere at 35 °C. ADH (0.22 g) and SS (0.31 g) were dissolved in *N*,*N*-dimethylformamide and slowly added into the flask. The mixture was stirred at 35 °C for 24 h. During this period, *N*,*N*-dimethylformamide was added to the flask to adjust the viscosity of the solution. After the reaction, methanol (3 mL) was added to the flask to consume the residual isocyanate. The viscous liquid was poured into the polytetrafluoroethylene mold and dried at 80 °C for 48 h. Finally, the binder D2000-ADH-SS was obtained.

### 2.3. Synthesis of D2000-ADH

First, D2000 (2.50 g) and IPDI (1.11 g) were put into a round bottom flask, subjected to magnetic stirring and left to react for 3 h in a nitrogen atmosphere at 35 °C. ADH (0.44 g) was dissolved in *N*,*N*-dimethylformamide and slowly added into the flask. The mixture was stirred at 35 °C for 24 h. During this period, *N*,*N*-dimethylformamide was added to the flask to adjust the viscosity of the solution. After the reaction, methanol (3 mL) was added to the flask to consume the residual isocyanate. The viscous liquid was poured into the polytetrafluoroethylene mold and dried at 80 °C for 48 h. Finally, the binder D2000-ADH was obtained.

### 2.4. Preparation of Energetic Composites

The polymer was dissolved in *N*,*N*-dimethylformamide; nano-Al powder was subsequently added to the polymer solution and stirred at 80 °C. After evaporating most of the solvent, the mixed slurry was dried at 80 °C for 24 h in a vacuum oven. It was cooled to room temperature to remove the lumps. Finally, the mixture was prepared as plate-shaped composites using a double-roll mill machine. The mass ratio of polymer to nano-aluminum powder was 20:80. According to the type of polymer, the composites were labeled as D2000-ADH-SS/Al, D2000-ADH/Al and F2603/Al, respectively.

### 2.5. Reprocessing of Adhesives and Energetic Composites

The binder or energetic composites fragments were placed directly on two steel plates. Then, we applied an external force of 9 MPa to the steel plates and heated them to 60 °C. After 0.5 h, they were left to cool to room temperature, and we took out the plate-like binder or energetic composites. The thickness was about 1 mm.

### 2.6. Characterization Methods

On a Bruker AVANCE III 500 MHz NMR spectrometer using DMSO-*d_6_* as solvent, ^1^H Nuclear Magnetic Resonance ^(^NMR) spectra were recorded. Fourier-transform infrared spectroscopy (FTIR) were recorded using a Bruker Tensor II spectrometer. X-ray diffraction (XRD) curves were recorded using an X-ray diffractometer (Bruker D8 Advance). Horiba labRAM ARAMIS Raman spectrum (wavelength 532 nm; range from 200 to 1500 cm^−1^) was collected to measure the characteristic peak of the -S-S- bond. The molecular weight of polymers was obtained from gel permeation chromatography (GPC) (Waters 1515). The temperature-dependent FTIR data were recorded using a Bruker Tensor II spectrometer, and the temperature interval was controlled at 10 °C in the range of 30~150 °C. Thermogravimetric analysis (TGA) curves were obtained using the Mettler 851e instrument with a heating rate of 10 °C min^−1^ from 40 to 600 °C in a nitrogen atmosphere. An atomic force microscope (AFM, Bruker Multimode 8) was employed to produce the polymer phase diagram. The small-angle X-ray scattering (SAXS) data were measured using a Bruker Nano STAR system, and Cu Kα radiation (λ = 0.1541 nm) was used as the X-ray source. Shimadzu AGS-X tester was used to measure the mechanical properties of polymers and energetic composites. The spline size was 3 × 1 cm^2^, and the tensile rate was 100 mm min^−1^. TA DMA Q800 was employed to dynamic mechanical behavior of polymers, and the dynamic strain was 0.01%. To further evaluate the reprocessing performance of binders and energetic composites, the fragments were hot pressed and measured using the Shimadzu AGS-X tester. The recovery rate was obtained by comparing the tensile strength and toughness. The stress recovery efficiency is the ratio of the stress of the sample after hot pressing to that of the initial sample, and the toughness recovery efficiency is the ratio of the toughness (the area of the tensile curve) of the sample after hot pressing to that of the initial sample.

## 3. Results and Discussion

### 3.1. Preparation and Characterization of D2000-ADH-SS Binder

Through a “one-pot two-step” reaction, D2000 reacted with IPDI to form a prepolymer, and then further reacted with ADH and SS mixture to generate the D2000-ADH-SS binder. The synthesis process is simple and controllable. For comparison, D2000-ADH was synthesized via a similar method. The detailed synthesis process is described in the Materials and Methods. In the FTIR curves (Figure 1a), the characteristic peaks at around 1095, 1549, 1641 and 3346 cm^−1^ correspond to ʋ(-C-O-C-), δ(-NH-), ʋ(-C=O) and ʋ(-NH-), respectively. The characteristic peak of isocyanate near 2600 cm^−1^ did not appear, indicating that the isocyanate completely reacted. To prove the chemical structure of D2000-ADH-SS (Figure S3), ^1^H NMR was employed. The aromatic hydrogen characteristic peaks were similar to those of 2-aminothiophenol that appeared in [29], revealing the successful implantation of disulfide. Raman spectra of D2000-ADH-SS (Figure S4) further proved the existence of disulfide bonds. D2000-ADH-SS had an obvious characteristic peak at around 485 cm^−1^, which corresponds to the vibration of the -S-S- bond. According to the GPC results (Table S1), the molecular weight of D2000-ADH-SS and D2000-ADH were 32.5 and 40.8 kDa, respectively, which may be attributed to the lower reactivity of SS than that of ADH. The polymer dispersion index (PDI) was around two, indicating a good molecular weight distribution [30]. When it was heated from 30 °C to 150 °C, the prominent characteristic peak of -NCO appeared near 2258 cm^−1^ in the FTIR curves (Figure S5), which was mainly due to the dynamic decomposition of ASCZ motifs [16]. The above characterization prove that D2000-ADH-SS binder was successfully synthesized.

**Figure 2:**
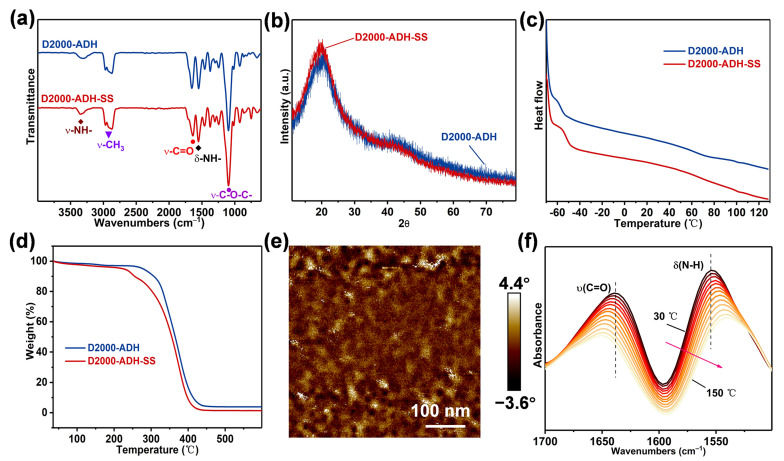
(**a**) FTIR of D2000-ADH and D2000-ADH-SS. (**b**) XRD, (**c**) DSC and (**d**) TGA curves of D2000-ADH and D2000-ADH-SS. (**e**) AFM phase image of D2000-ADH-SS. (**f**) Infrared spectra of D2000-ADH-SS at different temperatures from 1700 to 1500 cm^−1^.

XRD is often utilized to evaluate the crystallinity of polymers. There is no crystal peak in the XRD curves (Figure 2b), proving the amorphousness of polymers [31]. In the DSC curves (Figure 2c), the glass transition temperature (T_g_) of both polymers was lower than −50 °C, which was mainly due to the flexible polyether skeleton [32]. Low T_g_ values indicated that the polymers possessed good low-temperature resistance. There is no obvious exothermic peak in the DSC curves within the range from −60 to 120 °C, which further prove the amorphous nature of the polymers. The thermal properties of D2000-ADH-SS were evaluated by TGA. No obvious weight loss was observed below 230 °C (Figure 2d), indicating good thermal stability. Multiple hydrogen-bonded aggregates derived from ASCZ motifs could form tightly packed hard domain structures. Atomic force microscopy (AFM) was used to observe the microphase separation structures. D2000-ADH possesses a remarkable microphase separation structure, which was filled with a large number of hard domains (bright regions), which were mainly composed of multiple hydrogen bonds. Compared with D2000-ADH (Figure S6), D2000-ADH-SS had more loose, hard domains (Figure 2e). The SAXS curves were used to further compare the microphase separation structures of D2000-ADH and D2000-ADH-SS (Figure S7). After the zigzag disulfide structure was embedded in the polymer skeleton, the peak intensity and peak value decreased. The peak values of D2000-ADH and D2000-ADH-SS were 0.22 and 0.17 nm^−1^, respectively. According to the Bragg equation, the average domain spacings could be estimated to be 28.55 and 36.96 nm, which indicated that the embedding of disulfide compounds changed the microphase separation structures and formed more loose, hard domains [28]. One reason is that the zigzag structures broke the regular arrangement of hydrogen bonds, and the other reason is that the added disulfide could only form a quadruple hydrogen bond. After parts of ASCZ motifs were replaced by disulfides, the overall hydrogen bonding arrangement and hydrogen bonding density were significantly changed, thus affecting the hard domain structures. For the D2000-ADH-SS binder, with the increase in temperature, the stretching vibration peak of -C=O shifted to blue, and the characteristic peak of -NH- shifted to red, revealing the dissociation of dynamic hydrogen bonds. The above results prove that the implantation of disulfide with a special structure could change the hydrogen bond structures of polymer networks, realizing the optimization of dynamic adaptability and mechanical robustness.

### 3.2. Mechanical Properties of D2000-ADH-SS Binder

**Figure 3:**
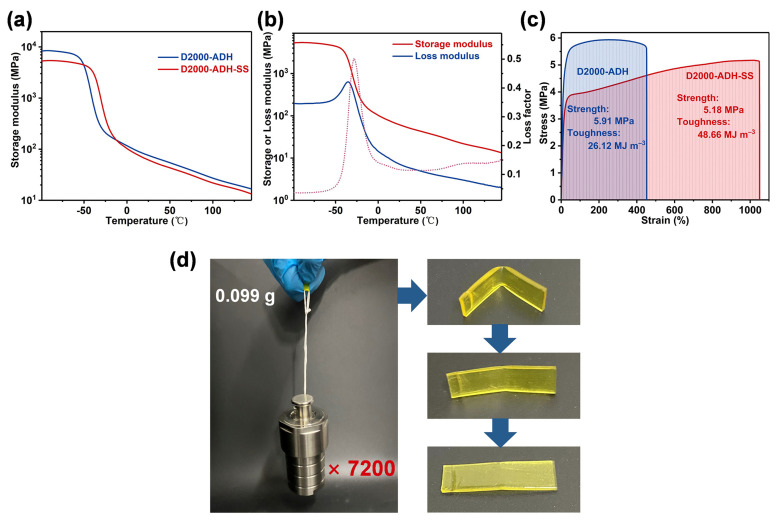
(**a**) Storage modulus curves of D2000-ADH and D2000-ADH-SS. (**b**) Storage and loss modulus curves of D2000-ADH. (**c**) Stress–strain curves of D2000-ADH and D2000-ADH-SS. (**d**) Optical images of D2000-ADH-SS, which may readily lift 7200 times its mass.

In energetic composites, polymer binders are mainly used to bond energetic crystals. Therefore, the mechanical properties of energetic composites are closely related to the polymer binders. The dynamic mechanical properties of polymers were evaluated by DMA. Initially, the storage modulus of D2000-ADH was higher than that of D2000-ADH-SS (Figure 3a), which was mainly due to the existence of denser hydrogen bond arrays in the former one. The storage modulus of D2000-ADH-SS began to decrease from −50 °C, indicating the phase transformation of the polymer initiated at this temperature. As shown in Figure 3b, the storage modulus and loss modulus of D2000-ADH-SS decreased with the increase in temperature. In the range of 0~150 °C, the storage modulus was greater than the loss modulus, indicating that D2000-ADH-SS had the properties of elastomer in this wide temperature range [20]. The tensile curves were used to further evaluate the mechanical properties of the polymers, and the results are shown in Figure 3c. With the effect of ASCZ motifs, D2000-ADH exhibited excellent mechanical properties (tensile strength: 5.91 MPa; toughness: 26.12 MJ m^−3^). Through the synergistic effect of disulfide, the toughness could be increased to 1.73 times, while the tensile strength just decreased by 0.73 MPa. The toughness of the D2000-ADH-SS allowed it to readily lift 7200 times its mass, and the good dynamic adaptability ensured that the spline could basically return to the initial state at room temperature within 6 h after removing the weight (Figure 3d). Here, the zigzag structures broke the original regular hydrogen bonding arrays and formed loose, hard domain structures. The activated hydrogen bonds could dissipate the strain energy through dissociation, and disulfide exchange was also beneficial to the rearrangement of entangled polymer chains. The activated hydrogen bonds and dynamic disulfide bonds promoted the slip of the polymer chain, which show that the ductility and toughness were significantly improved. This design strategy of improving the toughness and ductility of materials without excessive sacrifice of tensile strength provide a new idea for the molecular design of polymer binders.

### 3.3. Recycling Properties of D2000-ADH-SS Binder

**Figure 4:**
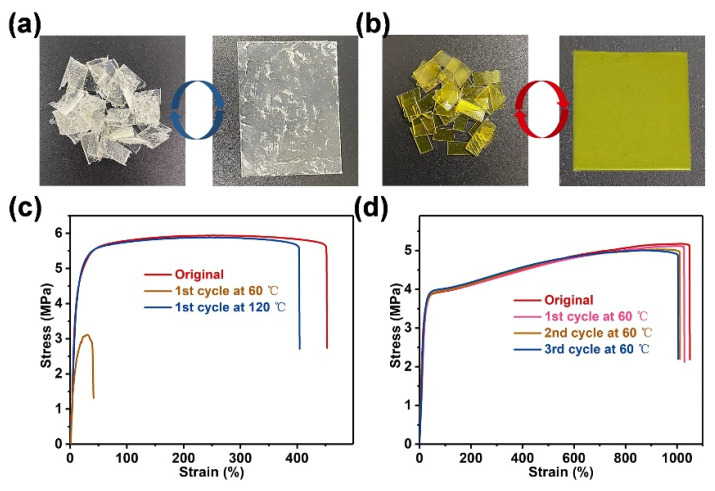
Photos of recycled (**a**) D2000-ADH and (**b**) D2000-ADH-SS. Tensile curves of recycled (**c**) D2000-ADH and (**d**) D2000-ADH-SS.

The reprocessing properties of polymer binders are mainly related to the dynamics of polymer networks. The ASCZ motifs endowed the polymer networks with mechanical robustness, but sacrificed the dynamic properties. Typically, the D2000-ADH fragments still had obvious cracks after hot pressing for 30 min at 60 °C (Figure 4a). When the materials were loaded with an external force, the stress concentrated at the crack, which accelerated the crack growth and seriously affected the mechanical properties. For energetic composite materials, the existence of cracks would also hinder the heat transfer, making the formation of “hot spots” at the cracks affect the safety of the weapon systems. The implantation of zigzag disulfide reduced the energy barrier of hydrogen bonding dissociation, and disulfide exchange further stimulated the rearrangement of the networks, eventually forming a more dynamic polymer network. Thanks to the good dynamic properties, D2000-ADH-SS fragments could be processed into smooth and complete plate-shaped materials under the same hot-pressing conditions (Figure 4b). The tensile curves in Figure 4c revealed that the existence of cracks lead to a serious reduction of the tensile properties of recycled D2000-ADH. Heating could reduce the energy barrier of hydrogen bonding dissociation, promoting the dynamic exchange of cross-linking points. When the hot-pressing temperature was raised to 120 °C, the tensile strength of D2000-ADH-SS could be restored to 5.81 MPa, but the side reaction of regenerated -NCO at a high temperature made the toughness significantly reduce [16]. In addition, a high temperature was not applicable to the processing sensitive energetic composites, and the safety needed to be further evaluated. In contrast, D2000-ADH-SS possesses excellent reprocessing abilities under the dual effects of zigzag structures and disulfide exchange. After the first hot pressing stage, the maximum tensile strength and toughness of D2000-ADH-SS were 5.09 MPa and 46.76 MJ m^−3^, respectively, which were 98.26% and 96.10% of the initial values (Figure 4d). Even after three cycles of hot-pressing processing, D2000-ADH-SS could still maintain 96.14% of the tensile strength and 94.14% of the toughness of the initial values, thus showing an excellent recycling performance. Therefore, the physical cross-linking networks constructed by ASCZ motifs and zigzag disulfide can not only enhance the mechanical properties of materials, but also improve the dynamic adaptability required for reprocessing. This strategy is also applicable to the design and preparation of recyclable materials.

### 3.4. Mechanical Properties and Reprocessing Performance of D2000-ADH-SS/Al

In order to cope with external impacts, collisions, shocks, etc., energetic composites need to possess good mechanical properties. At the same time, for energetic composite materials with cracks or defects, the traditional treatment method is destruction, which undoubtedly increases the economic losses and environmental pollution. The reprocessing of these damaged energetic composites is an economic and environmental treatment method. Because energetic crystals do not have plasticity, the dynamic properties of reprocessing mainly depend on polymer binders. In order to evaluate the effect of polymer binders on the mechanical properties and reprocessing performance of energetic composites, the designed polymer binder was used in the preparation and reprocessing process of energetic composites.

**Figure 5:**
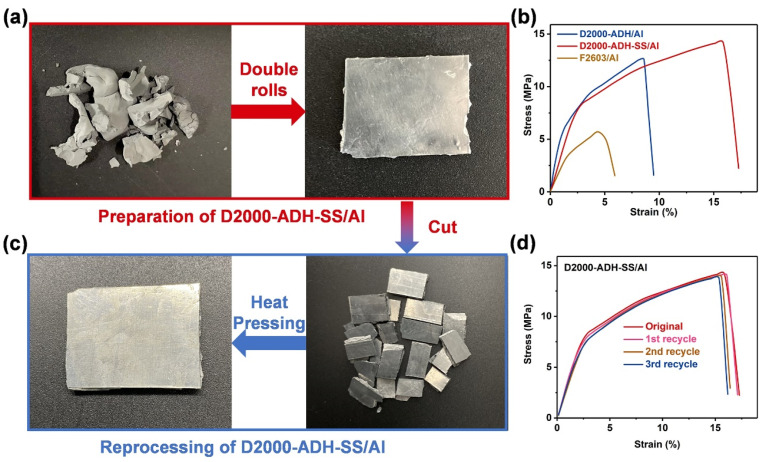
(**a**) Digital photos of D2000-ADH-SS/Al before and after processing. (**b**) Tensile curves of D2000-ADH-SS/Al, D2000-ADH/Al and F2603/Al. (**c**) Digital photos of D2000-ADH-SS/Al before and after reprocessing. (**d**) Tensile curves of recycled D2000-ADH-SS/Al.

D2000-ADH-SS/Al was included in a combination of 80 wt% nano-aluminum powder and 20 wt% polymers. Firstly, the polymers and nano-Al powder were mixed using a slurry method [33], and then pressed into plate-shaped composites at 60 °C with the double-roller mill (Figure 5a). D2000-ADH/Al and F2603/Al energetic composites were prepared by the same method. Figure 5b showed the mechanical properties of F2603/Al, D2000-ADH/Al and D2000-ADH-SS/Al. For the F2603/Al composite, the maximum tensile strength and toughness could reach 5.70 MPa and 0.22 MJ m^−3^, respectively. For the D2000-ADH/Al composite, the maximum tensile strength and toughness were 12.57 MPa and 0.84 MJ m^−3^, respectively. Compared with the commercial binder F2603, the supramolecular networks formed by multiple hydrogen bonds could significantly improve the tensile strength and toughness of energetic composites. After the zigzag disulfide compounds were implanted into the supramolecular networks, the mechanical properties were further improved. Among the energetic composites prepared with three kinds of binders, D2000-ADH-SS/Al was undoubtedly the most competitive one (maximum tensile strength: 14.19 MPa; toughness: 1.80 MJ m^−3^). The excellent mechanical properties were mainly due to the synergy of ASCZ motifs and zigzag disulfides.

Benefitting from the carefully designed dynamic networks, the shredded D2000-ADH-SS/Al could be restored to have a plate shape after just 30 min (hot-pressing conditions: 60 °C, 9 MPa) (Figure 5c). The surface of the plate-shaped D2000-ADH-SS/Al composite was smooth and complete, showing a good reprocessing performance. The D2000-ADH-SS/Al composites were then cut into rectangular splines and evaluated with a universal tensile machine. After the first hot-pressing cycle, the maximum tensile strength was restored to 14.08 MPa, and the toughness reached 1.77 MJ m^−3^, which were 99.22% and 98.33% of the original values, respectively (Figure 5d). With the increase in the cycle index, the recovered mechanical properties gradually decreased. However, even after three hot-pressing cycles, the strength and toughness of D2000-ADH-SS/Al were restored to 96.69% and 92.89% of the initial values, respectively. For D2000-ADH/Al, after just one hot-pressing cycle, the tensile strength and toughness reduced to 38.58% and 47.62% (Figure S8). The polymer network with dense hydrogen bonds restricted the dissociation of physical cross-links and the rearrangement of polymer chains, which is not conducive to the reprocessing of energetic composites. The zigzag structure may destroy the regular arrangement of hydrogen bonding arrays, and the disulfide exchange reaction could induce new “topological entanglement”, improving the integrity of the dynamic network after hot pressing. Thus, through these functions, the mechanical properties and reprocessing performance of energetic composites were significantly improved.

## 4. Conclusions

In conclusion, we proposed a novel molecular level design strategy for improving the mechanical properties and reprocessing properties of energetic composites. Firstly, multiple hydrogen bonds formed from ASCZ motifs were used to endow the polymer networks with mechanical robustness. The introduction of zigzag structures inhibited the regular arrangement of hydrogen bonds, thus promoting the dynamic exchange of hydrogen bonds. In addition, the disulfide exchange reaction further stimulated the rearrangement of polymer chains and improved the integrity of the network. As a result, the maximum tensile strength and toughness of the designed D2000-ADH-SS binder were 5.18 MPa and 48.66 MJ m^−3^, respectively. Compared with D2000-ADH polymer, the tensile strength just decreased by 0.73 MPa, but the toughness increased by 1.86 times.

The designed D2000-ADH-SS binder also endowed energetic composites with strong mechanical properties and excellent reprocessing abilities. Compared with the F2603/Al binder, D2000-ADH/Al increased the tensile strength and toughness to 2.21 and 3.82 times, respectively, indicating that ASCZ structures strengthened the physical cross-linking networks and improved the mechanical robustness. After the zigzag disulfides were implanted into the polymer networks, the mechanical properties and toughness of D2000-ADH-SS/Al composites reached 4.19 MPa and 1.80 MJ m^−3^, respectively. In addition, D2000-ADH-SS/Al also showed an excellent reprocessing performance. Even after three hot-pressing cycles, the recovered D2000-ADH-SS/Al composites still retained 96.69% of the stress and 92.89% of the toughness of the initial values, respectively. The proposed design strategy may provide a new idea for the processing and recycling of energetic composites, which greatly stimulate the practical application of energetic composites.

## Data Availability

All data generated or analyzed during this study are included in this published article (and its supplementary information files).

## Supplementary Materials

The following supporting information can be downloaded at: https://www.mdpi.com/article/10.3390/nano13061087/s1; Figure S1: Synthesis process of D2000-ADH-SS; Figure S2: Multiple hydrogen-bonded dimers of ASCZ; Figure S3: ^1^H NMR spectra of D2000-ADH-SS; Figure S4: Roman spectra of D2000-ADH-SS; Figure S5: Infrared spectra of D2000-ADH-SS at different temperature from 2275 to 2240 cm^−1^; Figure S6: AFM phase image of D2000-ADH; Figure S7: SAXS curves of D2000-ADH and D2000-ADH-SS; Figure S8: Tensile curves of recycled D2000-ADH/Al; Table S1: The molecular weights of D2000-ADH-SS and D2000-ADH.
