# Supplementary material for: Molecular Engineering of Binder for Improving the Mechanical Properties and Recyclability of Energetic Composites
Source: Nanomaterials (Basel). 2023 Mar 17;13(6):1087. doi: 10.3390/nano13061087 (PMC10051099; doi:10.3390/nano13061087)
Supplement: Supplementary file 1 [file nanomaterials-13-01087-s001.zip › nanomaterials-2280929-supplementary.pdf]

Molecular engineering of binder for improving the mechanical properties and recyclability of energetic composites

Jing Yang[1], Xin Zhou[1], Xiaomu Wen[2], Gazi Hao[1], Lei Xiao[1], Guangpu Zhang[1,*], and Wei Jiang[1,*]

[1] National Special Superfine Powder Engineering Technology Research Center, Nanjing University of Science and Technology, Nanjing 210094, PR China; yjnjut126@126.com (J.Y.); 18851965918@163.com (X.Z.); hgznjust1989@163.com (G.H.); superfine_xiaolei@njust.edu.cn (L.X.);

[2] Science and Technology on Transient Impact Laboratory, Research Institute of China Ordnance Industries, Beijing 102202, China; wenxm2908@163.com (X.W.)

* Correspondence: gpzhang@njust.edu.cn (G.Z.), superfine_jw@126.com (W.J.)

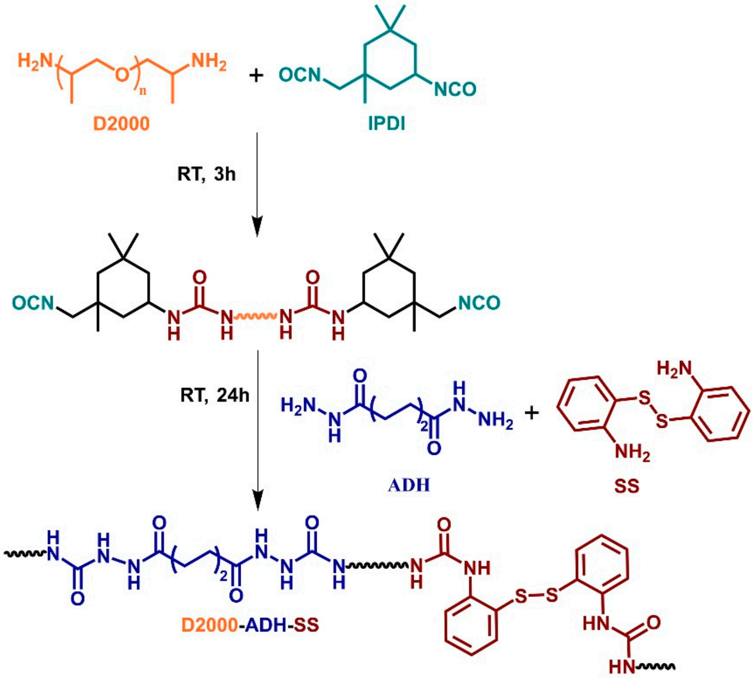

Figure S1. Synthesis process of D2000-ADH-SS.

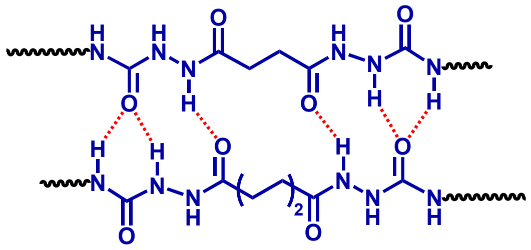

Figure S2. Multiple hydrogen-bonded dimers of ASCZ.

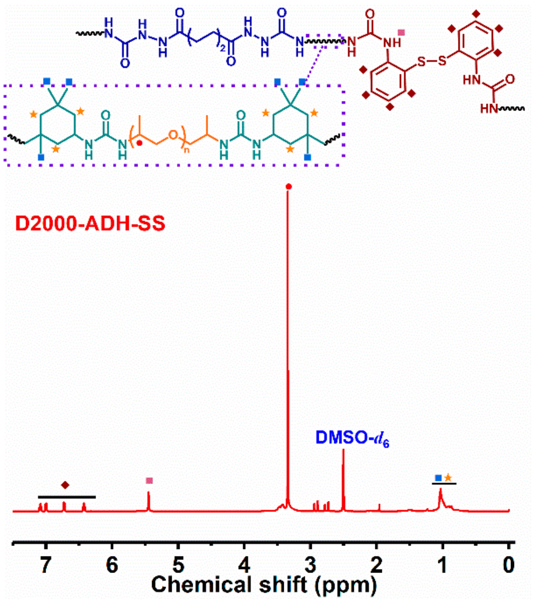

Figure S3. ^{1}H NMR spectra of D2000-ADH-SS.

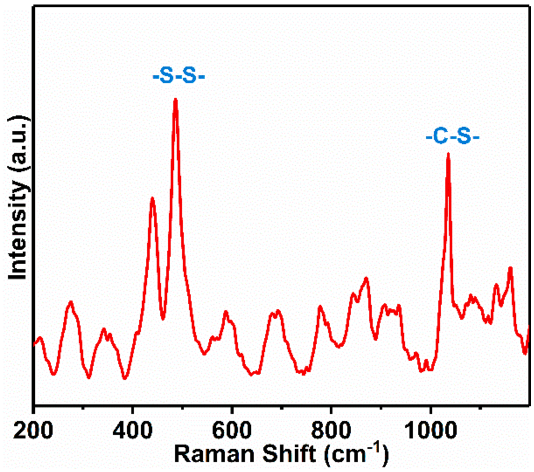

Figure S4. Roman spectra of D2000-ADH-SS.

Table S1. The molecular weight of D2000-ADH-SS and D2000-ADH.

	Mn	Mw	PDI
D2000-ADH	40804	81290	1.99
D2000-ADH-SS	32465	77806	2.40

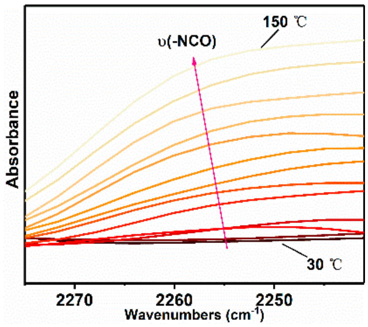

Figure S5. Infrared spectra of D2000-ADH-SS at different temperature from 2275 to 2240 cm^{-1}.

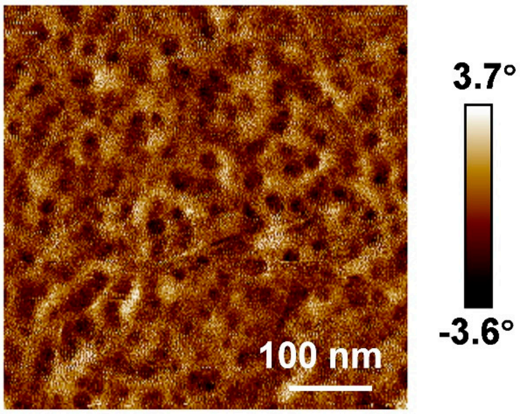

Figure S6. AFM phase image of D2000-ADH.

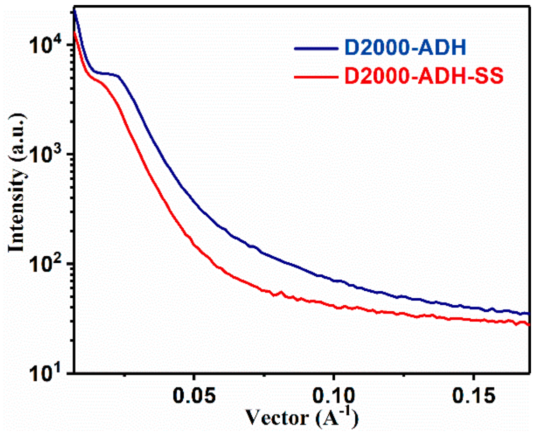

Figure S7. SAXS curves of D2000-ADH and D2000-ADH-SS.

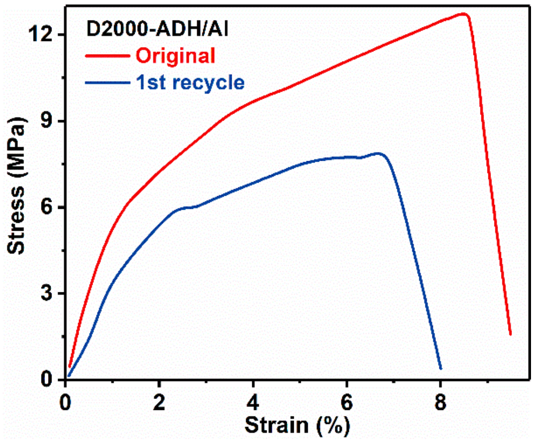

Figure S8. Tensile curves of recycled D2000-ADH/Al.
